# Linac‐ and CyberKnife‐based MRI‐only treatment planning of prostate SBRT using an optimized synthetic CT calibration curve

**DOI:** 10.1002/acm2.14411

**Published:** 2024-06-04

**Authors:** Jessica Scholey, Tomi Nano, Kamal Singhrao, Osama Mohamad, Lisa Singer, Peder Eric Zufall Larson, Martina Descovich

**Affiliations:** ^1^ Department of Radiation Oncology University of California San Francisco San Francisco California USA; ^2^ Department of Radiology and Biomedical Imaging University of California San Francisco San Francisco California USA

**Keywords:** magnetic resonance imaging, prostate cancer, radiotherapy, synthetic CT, treatment planning

## Abstract

**Purpose:**

CT Hounsfield Units (HUs) are converted to electron density using a calibration curve obtained from physical measurements of an electron density phantom. HU values assigned to an MRI‐derived synthetic computed tomography (sCT) may present a different relationship with electron density compared to CT HU. Correct assignment of sCT HU values is critical for accurate dose calculation and delivery. The goals of this work were to develop a sCT calibration curve using patient data acquired on a clinically commissioned CT scanner and assess for CyberKnife‐ and volumetric modulated arc therapy (VMAT)‐based MR‐only treatment planning of prostate SBRT.

**Methods:**

Same‐day CT and MRI simulation in the treatment position were performed on 10 patients treated with SBRT to the prostate. Dixon in‐phase and out‐of‐phase MRIs were acquired on a 3T scanner using a 3D T1‐weighted gradient‐echo sequence to generate sCTs using a commercial sCT algorithm. CT and sCT datasets were co‐registered and HU values compared using mean absolute error (MAE). An optimized HU‐to‐density calibration curve was created based on average HU values across an institutional patient database for each of the four sCT tissue types. Clinical CyberKnife and VMAT treatment plans were generated on each patient CT and recomputed onto corresponding sCTs. Dose distributions computed using CT and sCT were compared using gamma criteria and dose‐volume‐histograms.

**Results:**

For the optimized calibration curve, HU values were −96, 37, 204, and 1170 and relative electron densities were 0.95, 1.04, 1.1, and 1.7 for adipose, soft tissue, inner bone, and outer bone, respectively. The proposed sCT protocol produced total MAE of 94 ± 20HU. Gamma values mean ± std (min‐max) were 98.9% ± 0.9% (97.1%–100%) and 97.7% ± 1.3% (95.3%–99.3%) for VMAT and CyberKnife plans, respectively.

**Conclusion:**

MRI‐derived sCT using the proposed approach shows excellent dosimetric agreement with conventional CT simulation, demonstrating the feasibility of MRI‐derived sCT for prostate SBRT treatment planning.

## 1 INTRODUCTION

Prostate cancer is the leading cause of cancer incidence in men in the United States with an estimated 268,000 diagnoses in 2022.^1^ Radiotherapy is a non‐invasive option for managing prostate cancer that provides high success rates for curing the disease.^2^ Patients being treated with stereotactic body radiotherapy (SBRT) to the prostate routinely undergo magnetic resonance imaging (MRI) for purposes of anatomical delineation. The soft tissue contrast provided by MRI is particularly well suited for visualizing prostatic lesions and critical structures such as the urethra^3^, ^4^ while also decreasing interobserver variability^5^ versus computed tomography (CT) alone.

In the radiotherapy workflow, patients often receive both an MRI (for anatomical visualization) and CT (for density information used in dose calculation).^6^ Replacing CT with MRI‐based synthetic CT (sCT) for dose calculation would offer many advantages such as increased efficiency, reduction in the number of imaging procedures required for each patient, and a decrease in uncertainties related to multi‐modality image registration.^7^ While MR images demonstrate favorable qualities for the delineation of many normal and diseased tissue types, they do not provide a direct map of photon attenuation, hindering its utility for dose calculation. Over the past decade there has been increased research in generating sCTs from MRIs using atlas‐based, deep learning‐based or hybrid techniques.^8^, ^9^, ^10^ However, clinical implementation has been slow to develop as there are currently no consensus guidelines or recommendations provided for using MRI‐derived sCTs in practice. A major challenge is that, unlike with Hounsfield Units (HUs) of CT scans, HUs of MRI‐derived sCTs do not correspond directly to physical values of photon attenuation and improved methods of HU mapping and benchmarking during sCT generation has been called for in the literature.^8^, ^11^ In radiation therapy planning, CT HU are converted to electron density by means of a calibration curve obtained from physical measurements of an electron density phantom. The HU assigned to a sCT may present a different relationship with electron density, compared to CT HU. Therefore, using the clinically adopted calibration curve for CT images might lead to errors in dose calculations.^12^ As it is not possible to generate sCT specific electron density calibration curves by means of physical measurements, assignment of HU values consistent with those obtained from CT images for each tissue type is critical for accurate dose calculation and delivery.^13^

The goal of this work was to develop a CT calibration curve optimized for sCT dose calculation using patient data acquired on a clinically commissioned CT scanner and implement with a commercially available MRI‐derived sCT protocol. Treatment planning for two common modalities used to treat prostate cancer with SBRT were investigated, namely, volumetric modulated arc therapy (VMAT) delivered on a conventional C‐arm linear accelerator and the CyberKnife system (Accuray, Sunnyvale, CA, USA), a compact linear accelerator mounted onto a robotic base.

## 2 MATERIALS AND METHODS

### 2.1 Imaging datasets

Ten patients being treated with SBRT to the prostate were prospectively recruited under this IRB‐approved study. Same‐day CT and MRI simulation in the head‐first supine treatment position were performed on each patient. CT scans were acquired at 120 kVp photon energy using a helical acquisition with reconstructed slices of voxel size 1 × 1 × 1.5 mm^3^ (SOMATOM Definition AS, Siemens Healthcare, Erlangen, Germany). MRI simulation scans were acquired on a three Tesla scanner (MAGNETOM Vida, Siemens Healthcare, Erlangen, Germany) using a radiotherapy‐dedicated indexed couch overlay and large 18‐channel UltraFlex coil (Siemens Healthcare, Erlangen, Germany) suspended on a coil bridge to prevent deformation of the patients’ surface caused by the coil. Dixon in‐phase and out‐of‐phase images were acquired using a 3D T_1_‐weighted volumetric interpolated breath‐hold examination (VIBE) sequence (TE_1_/TE_2_/TR = 1.23/2.46/4 ms, readout bandwidth = 1090 Hz/pixel, 1 × 1 × 1.5 mm^3^, 176−224 slices, scan time 5 min). sCT datasets were generated using a vendor‐provided algorithm based on in‐phase, out‐of‐phase, fat‐only, and water‐only MRIs (syngo.via RT Image Suite, Siemens Healthcare, Erlangen, Germany). CT and sCT datasets were co‐registered in MIM Software (MIM Software Inc, v 6.8.3 Cleveland, OH, USA) using the automated 3D rigid registration algorithm used clinically at our institution.

### 2.2 Synthetic CT reconstruction algorithm

An FDA‐approved hybrid algorithm for generating sCT images from MRI in the pelvis was used to generate sCTs using a combination of tissue classifiers and anatomic atlases.^14^, ^15^ The protocol requires acquisition of a set of Dixon in‐phase and out‐of‐phase images in the axial plane using a T_1_‐weighted VIBE sequence and automatic 3D distortion correction. The sCT algorithm classifies material as air, adipose, soft tissue, inner bone, or outer bone corresponding to HU values of −1000, −75, 0, 204, and 1170, respectively. In this algorithm, tissue and adipose are classified using spectral information, air using thresholding, and bone using a multi‐atlas‐based model based on the in‐phase, out‐of‐phase, fat‐only, and water‐only MRIs produced by the VIBE Dixon sequence.

### 2.3 Comparison between synthetic and real CTs

To compare sCT and CT HU values for the ten datasets evaluated, air, adipose, soft tissue, inner bone, and outer bone were segmented on each patients’ CT dataset. Intensity‐based thresholding was used to define each region, with a final mask created for each tissue type based on the initial thresholding and manual contouring in MIM. For each patient, the masks were used to define corresponding structures representing adipose, soft tissue, inner bone, and outer bone. HU values (mean and standard deviation) were computed and averaged across all patient CT datasets.

For visualization, difference HU maps were computed by subtracting the sCT from its corresponding co‐registered CT. HU values of sCTs and CTs were compared using mean absolute error (MAE) across all imaging voxels *N* according to the following equation:

(1) $$\mathrm{MAE}=\frac{1}{N}\sum_{i=1}^{N}\left|sCT(i)-CT(i)\right|$$

where, sCT(i) and CT(i) are HU values of the $i^{th}$ voxel in the co‐registered sCT and CT, respectively.

### 2.4 sCT calibration curve and radiotherapy treatment planning

In radiation therapy, calibration curves relating CT HU with electron density are typically generated using vendor‐provided tissue substitute phantoms containing plugs of known density values.^16^ Performing this task for sCT HU calibration curves is not straightforward as it requires anthropomorphic phantoms made of materials with tissue‐like contrast in both CT and MRI,^17^ which are not commercially available. Therefore, two calibration curves relating sCT HU with relative electron density were generated: one using the original sCT HU values (“original sCT curve”) and another using optimized HU values (“optimized sCT curve”). The optimized sCT curve was created using mean HU values computed for adipose, soft tissue, inner bone, and outer bone averaged across all patient CTs. For purposes of treatment planning, both calibration curves were input into RayStation v7.0 (RaySearch Medical Laboratories AB, Stockholm, Sweden) and Accuray Precision v3.1 (Accuray, Sunnyvale, CA) treatment planning systems.

VMAT treatment plans were generated in RayStation using a two 6 MV coplanar arc arrangement and dose calculation performed with a collapsed cone dose calculation algorithm. CyberKnife treatment plans were generated in Precision using a 6FFF non‐isocentric beam arrangement and dose calculation performed with a ray‐tracing dose calculation algorithm. All plans were generated according to institutional practice and met clinical objectives. For each patient, treatment plans were generated on CT datasets and recomputed onto corresponding sCT datasets using identical plan parameters. For comparison, dose calculation was performed using both the original and the optimized sCT calibration curves. Dose distributions were compared using gamma analysis (3%/3 mm local dose threshold) and dose‐volume‐histograms (DVHs) of target and critical structures including the bladder, rectum, femoral heads, large bowel, and small bowel.

## 3 RESULTS

### 3.1 Derivation of sCT calibration curve

**FIGURE 1:**
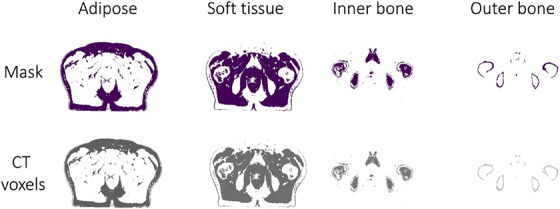
CT‐derived segmentation of adipose, soft tissue, inner bone, and outer bone in an example patient. CT HU values (mean and standard deviation) were compared to sCT HU values. CT, computed tomography; HU, Hounsfield Unit; sCT, synthetic CT.

Example masks for adipose, soft tissue, inner bone, and outer bone segmented using intensity‐based thresholding is shown in the top row of Figure 1. For each patient, the masks were used to define corresponding regions representing adipose, soft tissue, inner bone, and outer bone in the CT datasets as shown in the bottom row of Figure 1.

HU values for air, adipose, soft tissue, inner bone, and outer bone provided in the literature,^18^ extracted from patient CT datasets, and MRI‐generated sCT datasets are tabulated in Table 1.

**TABLE 1:** Tabulated HU values for air, adipose, soft tissue, inner bone, and outer bone provided in the literature,^18^ extracted from CT datasets, and original MRI‐derived sCT datasets.

	Literature	CT	Original sCT
Air	−1000	−1000	−1000
Adipose	−100	−96 ± 6.1	−75
Soft tissue	30–45	37 ± 3.9	0
Inner bone	200–800	219 ± 14	204
Outer bone	>1000	1000 ± 23	1170

Abbreviations: CT, computed tomography; HU, Hounsfield Unit; sCT, synthetic CT.

Of note, original sCT HU values for adipose and soft tissue disagree with those for CT and are outside the range of values provided in the literature. Furthermore, adipose and soft tissue structures typically make up the largest volume of the pelvis datasets, as shown in Figure 1, and mischaracterization of these HU values could lead to incorrect effective beam path lengths computed in the dose calculation algorithms.

**FIGURE 2:**
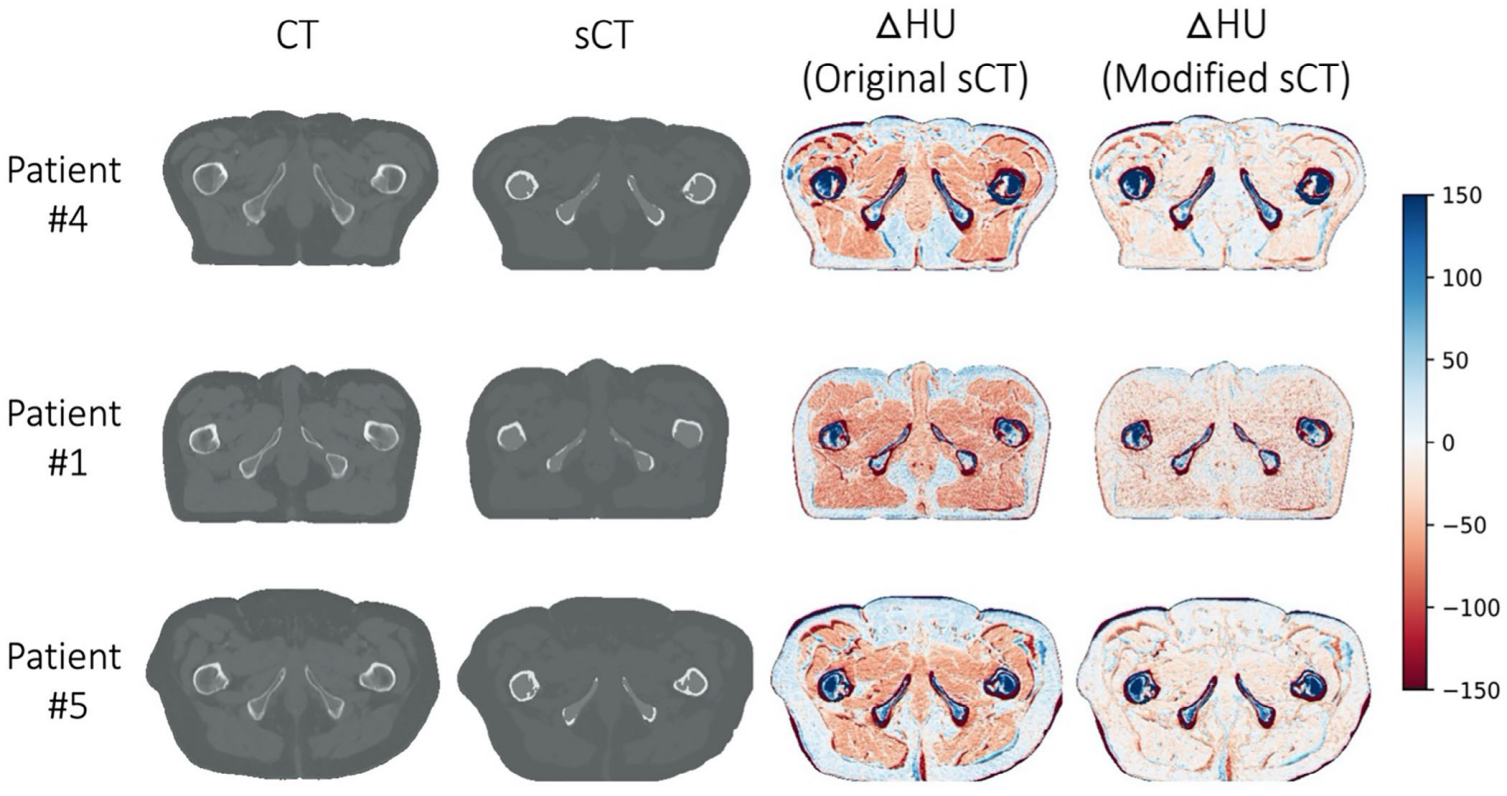
Three exemplary datasets of CT (first column), sCT (second column), the original HU difference map (third column), and a modified HU difference that was created by manually overriding the sCT values for adipose and soft tissue to match the CT (fourth column). HU values are displayed from −150 to 150 to better visualize the differences in seen soft tissue and fat. CT, computed tomography; HU, Hounsfield Unit; sCT, synthetic CT.

HU difference maps are illustrated in Figure 2, which shows three exemplary datasets of CT (first column), sCT (second column), the original HU difference map (third column), and a modified HU difference map that was created by manually overriding the sCT values for adipose and soft tissue to match the CT (fourth column). In other words, in the modified sCT HU difference map, the sCT classification for air, adipose, soft tissue, inner bone or outer bone corresponded to HU values of −1000, −96 (instead of −75), 37 (instead of 0), 204, and 1170, respectively. As demonstrated in column D, these modified sCT datasets agreed more closely with CT datasets in soft tissue and adipose regions.

As expected, by replacing HU values for soft tissue and fat in the sCT with the average values estimated from the CT, the total MAE (mean and standard deviation amongst all 10 datasets) was reduced from 106.9 ± 17.9 to 93.9 ± 20.1 HU for the original versus modified sCT, respectively. The soft tissue MAE was reduced from 76.5 ± 7.6 to 50.8 ± 8.1 HU when calculated for the original versus modified sCT, respectively. The adipose MAE was reduced from 96.2 ± 30.2 to 93.6 ± 32.2 HU when calculated for the original versus modified sCT, respectively. Because original sCT values for bone were within the range reported in the literature, this HU value was not modified in the sCT datasets, and the total bone MAE is reported to illustrate the variation among patients. MAE HU results for each patient in the study are reported in Table 2.

**TABLE 2:** Tabulated values of MAE (HU) for HU difference maps using the original sCT versus the modified sCT for the patients analyzed for this study.

	Total MAE (HU)		Soft tissue MAE (HU)		Adipose MAE (HU)		Total bone MAE (HU)
Patient #	Original	Modified	Original	Modified	Original	Modified	Original only
1	77.8	62.6	73.7	45.5	48.2	43.2	232.5
2	94.0	79.4	74.0	46.4	68.4	63.0	319.8
3	136.5	127.6	71.9	50.6	147.4	150.9	367.5
4	126.2	112.2	90.6	62.4	120.4	117.7	314.4
5	113.4	101.2	85.6	58.9	111.1	108.7	311.5
6	117.8	111.5	83.1	64.0	113.0	113.7	334.9
7	102.4	86.1	75.9	47.8	84.4	80.3	329.4
8	112.5	98.5	72.7	46.8	113.8	106.7	309.4
9	87.5	72.0	64.9	39.7	66.2	62.5	315.8
10	100.6	87.7	72.7	45.7	88.9	89.3	321.1

Abbreviations: CT, computed tomography; HU, Hounsfield Unit; sCT, synthetic CT.

**FIGURE 3:**
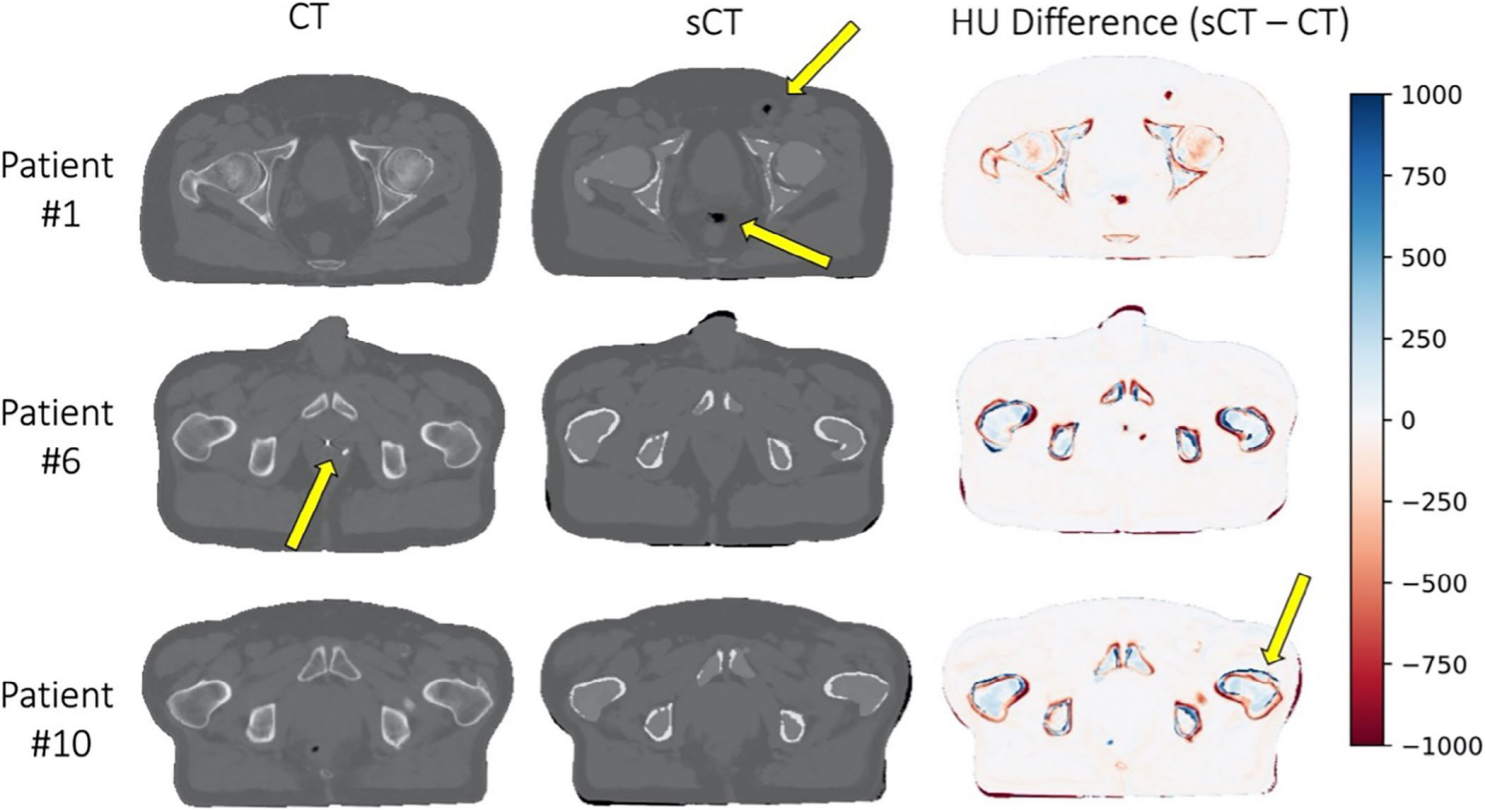
Example axial CT (left), sCT (middle), and HU difference maps (right) for three patients included in this study. CT, computed tomography; HU, Hounsfield Unit; sCT, synthetic CT.

Areas of disagreement most commonly seen between sCT and CT datasets are depicted in Figure 3. As expected, differences could be seen in instances of variable bowel and/or rectal gas filling due to anatomical changes between image acquisition (top row). Gold fiducial makers placed in the prostate for purposes of image alignment or calcifications within the prostate gland appeared as hyperintense on CT and hypointense on MRI (therefore not appearing in sCTs), resulting in additional discrepancies seen between datasets (middle row). Misalignment of bone due to small differences in pelvic flexion and/or leg positioning was also occasionally seen (bottom row).

**FIGURE 4:**
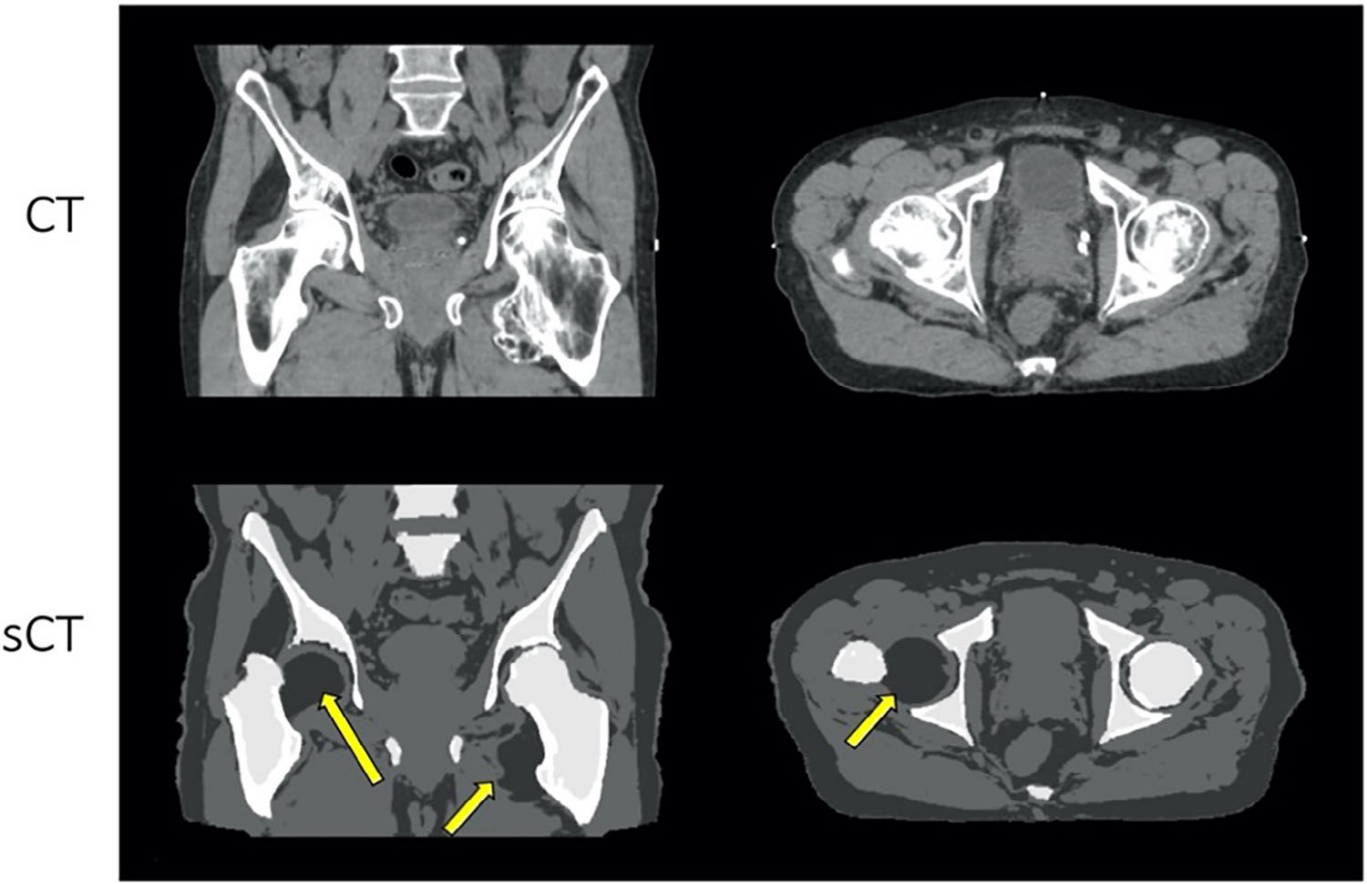
Example sagittal and axial slices of CT (top row) and sCT (bottom row) datasets for Patient #9 whose datasets exhibited the largest deviation in bone segmentation, which may have been caused by the abnormal shape of the left femoral bone which challenges atlas‐based reconstruction. CT, computed tomography; sCT, synthetic CT.

Mislabeling of bone remains an ongoing challenge when generating CT from MRI due to minimal signal being produced by bone in most clinical MRI sequences. The most drastic deviation was seen in one patient whose right femoral head was displaced (shown in Figure 4), which may have been caused by the abnormal shape of the left femoral bone in this patient that creates challenges for atlas‐based reconstruction.

**FIGURE 5:**
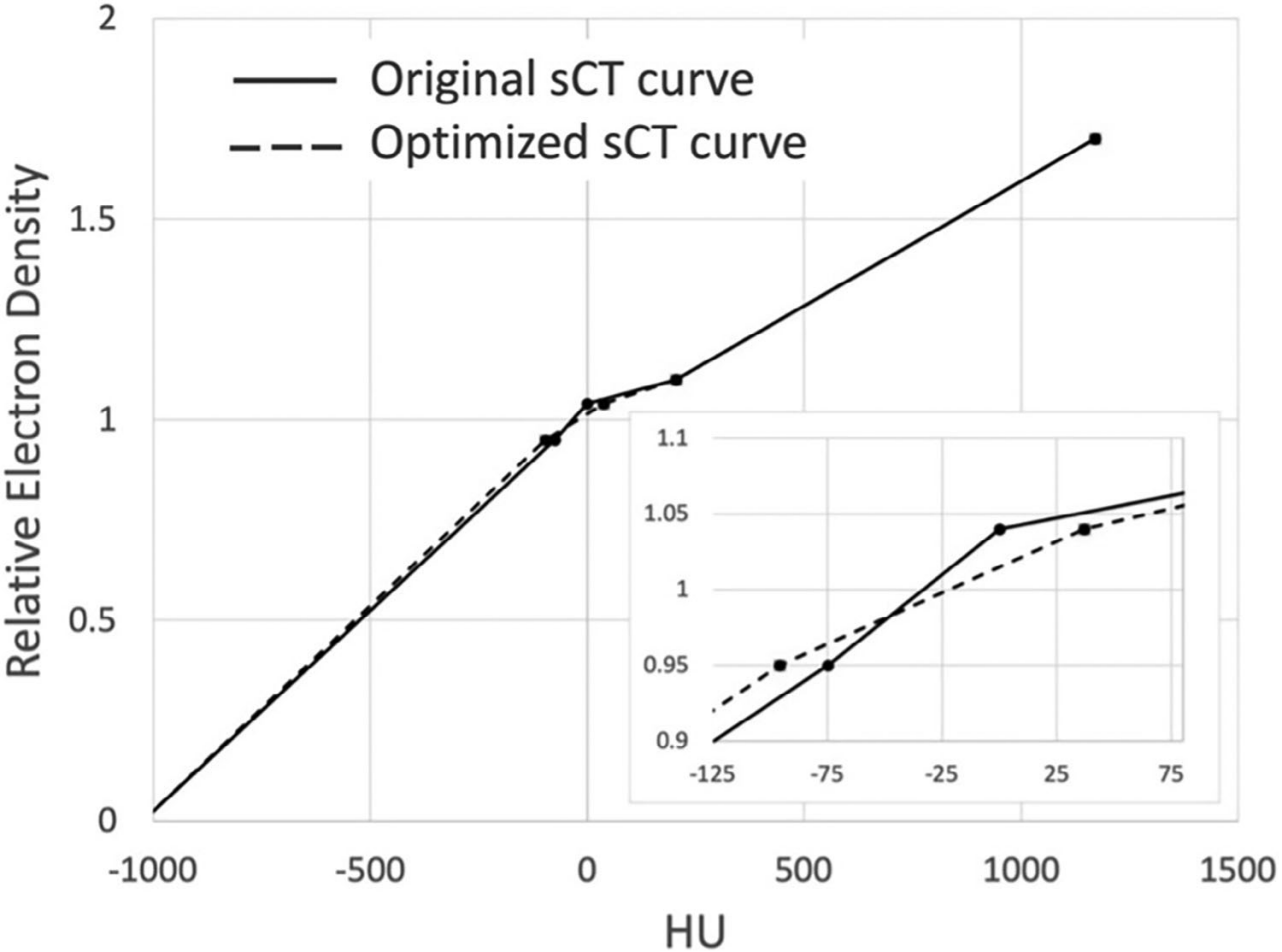
Original (solid line) and optimized (dashed line) sCT calibration curves used for dose calculation in the clinical treatment planning systems. sCT, synthetic CT.

For purposes of dose calculation in the treatment planning systems, an optimized CT calibration curve was generated using HU versus relative electron density values for air, adipose, soft tissue, inner bone, and outer bone, respectively. The calibration curve was generated using HU values computed for each tissue type averaged across the 10 prostate patients included in this study, as shown in Table 1. An illustration of the optimized sCT calibration curve derived in this work (with the original sCT curve for comparison) is shown in Figure 5.

### 3.2 Treatment plan evaluation

**FIGURE 6:**
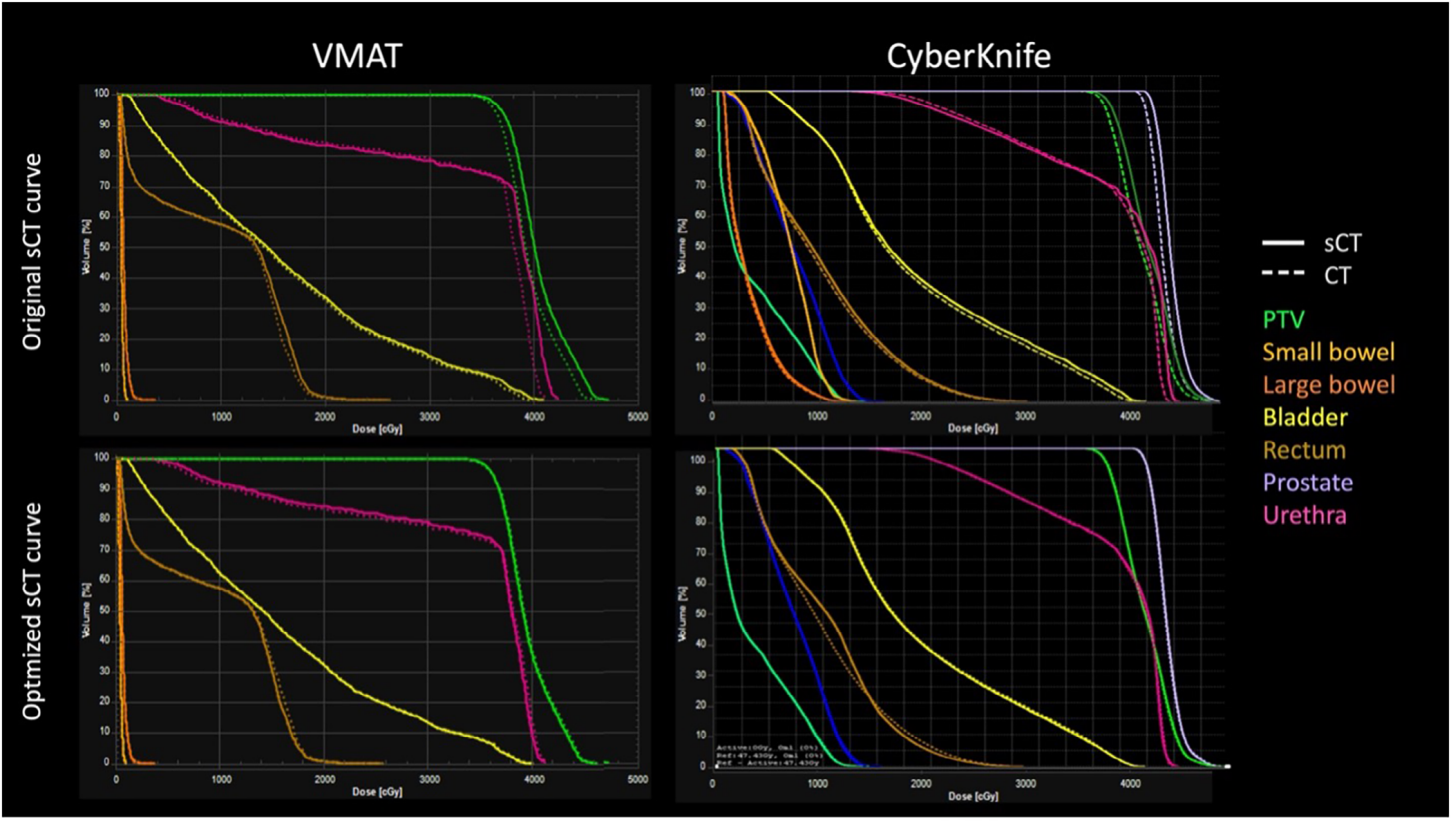
Example DVH plots in sCT (solid line) versus CT (dashed line) datasets demonstrating the systematic discrepancies when using the original CT calibration curve (top row) which are resolved when implementing the optimized CT calibration curve (bottom row) for VMAT (left column) and CyberKnife (right column) treatment plans. CT, computed tomography; DVH, dose‐volume‐histogram; sCT, synthetic CT; VMAT, volumetric modulated arc therapy.

Results for all plans calculated using the original (non‐optimized) sCT calibration curve demonstrated systematic discrepancies between DVHs in sCT versus CT datasets, as shown by non‐coincident histograms in the top row of Figure 6. This is caused by inaccurate scaling of each beamlets’ effective path length caused by incorrect HU values assigned to soft tissue and adipose. However, these discrepancies were resolved when the optimized sCT calibration curve was used, as shown by coincident histograms in the bottom row of Figure 6.

Dose distributions for treatment plans created using the optimized sCT calibration curve were compared by calculating the gamma metric using 3%/3 mm local criteria. Gamma metric results (mean, standard deviation and min‐max amongst all datasets) were 98.9 ± 0.9% (97.1%–100%) and 97.7 ± 1.3% (95.3%–99.3%) for VMAT and CyberKnife plans, respectively. All gamma results were > 95%, indicating good dosimetric agreement between plans calculated on sCT versus CT datasets. Dose to 95% of planning target volume (PTV) in sCT plans received 100.5% ± 0.8% (99.8%–102.5%) and 97.0% ± 6.0% (81.9%–103.0%) of the PTV volume in the CT plans for VMAT and CyberKnife plans, respectively. All values of 95% PTV coverage for sCT datasets were within 4% of corresponding CT datasets, with the exception of Patient #9 whose PTV in the CyberKnife plan received lower dose coverage in the sCT dataset versus CT dataset due to the mislabeling of the bone/femoral head as previously described. Gamma metrics and dose to 95% of PTV volumes for each patient are shown in Table 3.

**TABLE 3:** Gamma metrics and relative PTV coverage for plans calculated on VMAT and CyberKnife plans for the patients evaluated in this study.

	Gamma metric (3%/3 mm)		Dose to 95% of PTV (sCT relative to CT)	
Patient #	VMAT	CyberKnife	VMAT	CyberKnife
1	99.96	99.27	100.1	98.8
2	99.39	96.02	102.5	96.6
3	97.96	98.91	101.0	98.5
4	99.63	98.60	100.1	99.4
5	99.13	97.26	100.4	101.7
6	97.13	95.27	100.1	98.3
7	99.05	98.56	100.2	99.0
8	98.00	96.5	100.5	103.0
9	99.71	98.01	99.8	81.9
10	98.91	98.28	100.6	93.0

**FIGURE 7:**
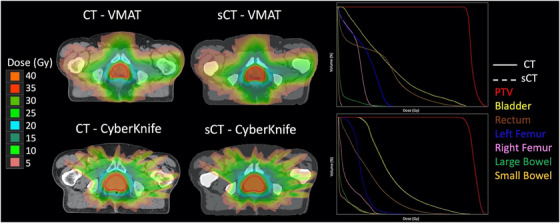
Representative axial dose distributions for VMAT (top row) and CyberKnife (bottom row) plans calculated on a CT (left images) versus sCT (right images). DVH curves in each plot (right column) directly overlay one another, demonstrating close agreement between distributions for sCT versus CT when the optimized sCT calibration curve is used. CT, computed tomography; DVH, dose‐volume‐histogram; sCT, synthetic CT; VMAT, volumetric modulated arc therapy.

Representative axial dose distributions for VMAT (top row) and CyberKnife (bottom row) plans calculated on a CT (left images) versus sCT (right images) when using the optimized calibration curve are shown in Figure 7. Close agreement between DVHs for sCT versus CT can be seen for both plan types, demonstrated by the indistinguishable dose volume histograms.

## 4 DISCUSSION

In this work, an MRI‐derived sCT protocol was optimized and assessed for treatment planning on a dataset of ten patients treated for prostate cancer using VMAT and CyberKnife. sCT and CT HU values were compared, and an optimized calibration curve was generated to provide better agreement between sCT HU values and treatment plan dose distributions calculated on sCT versus CT datasets. Our findings were consistent with previous work for MRI‐based dose calculation for prostate SBRT^12^ and is the first to evaluate the feasibility of MRI‐only treatment planning for robotic radiosurgery.

HU values for soft tissue and adipose in the original sCT datasets demonstrated disagreement with values from the literature and estimated from CT. For example, the HU value used for soft tissue was 0 HU, rather than a more typical value of 30−45 HU. Similarly, the HU value for adipose in the original sCT was −75 HU, versus values around −100 HU typically seen in fatty tissues. Manually overriding the sCT values for adipose and soft tissue to match the CT resulted in better agreement between sCT and CT. The total MAE was reduced from 106.9 ± 17.9 to 93.9 ± 20.1 HU for the original versus modified sCT, respectively. The soft tissue MAE was reduced from 76.5 ± 7.6 to 50.8 ± 8.1 HU when calculated for the original versus modified sCT, respectively. The adipose MAE was reduced from 96.2 ± 30.2 to 93.6 ± 32.2 HU when calculated for the original versus modified sCT, respectively. Overall, these MAE values are consistent with those reported in the literature for atlas‐based MRI‐derived sCT.^19^

In clinical practice, it is not uncommon to perform manual HU overrides of materials in the treatment planning systems to better match the planning datasets to a patients’ true anatomy. For example, this is routinely performed in cases where CTs exhibit artifacts caused by high‐Z materials like dental implants,^20^ when a portion of the patient anatomy is cut off due to insufficient field‐of‐view^21^ due to imaging a patient with large body habitus, or when metallic implants saturate the CT number scale and an appropriate HU value must be manually assigned according to the implant material.^22^ In the case of sCT, mitigating HU value differences can similarly be performed by manually assigning HU values of the sCT to better match CT values. However, performing this manual override on each patient dataset in practice may result in logistical challenges related to clinical streamlining, the potential need for image post‐processing to perform manual overrides, and associated quality assurance measures required to validate these modifications on a per‐patient basis. To circumvent these challenges, the same dosimetric outcome can be achieved by generating a global sCT‐specific CT calibration curve derived from a local patient database, as described in this work.

In this study, disagreement could be seen in instances of changing bowel/rectal gas filling between scans, fiducials or calcifications appearing in the CT but not in the sCT, and/or bone density distribution differences. Accurate bone delineation remains an ongoing challenge for reconstruction of MRI‐based sCT, with a single outlier CyberKnife plan yielding a significant under‐dose of the target volume (D_95% _= 81.9%) occurring due to a mischaracterization of a femoral head as soft tissue. Mislabeling of bone can be problematic for treatment planning even when the sCT calibration curve is used. Recent advances in ultrashort echo time (UTE) or zero echo time (ZTE) MRI utilize very short echo times capable of acquiring signal in bone.^23^, ^24^, ^25^ While these sequences are not yet acquired routinely, they show great promise for better distinguishing between bone and air^26^ and will likely be increasingly implemented for this purpose.^27^

**FIGURE 8:**
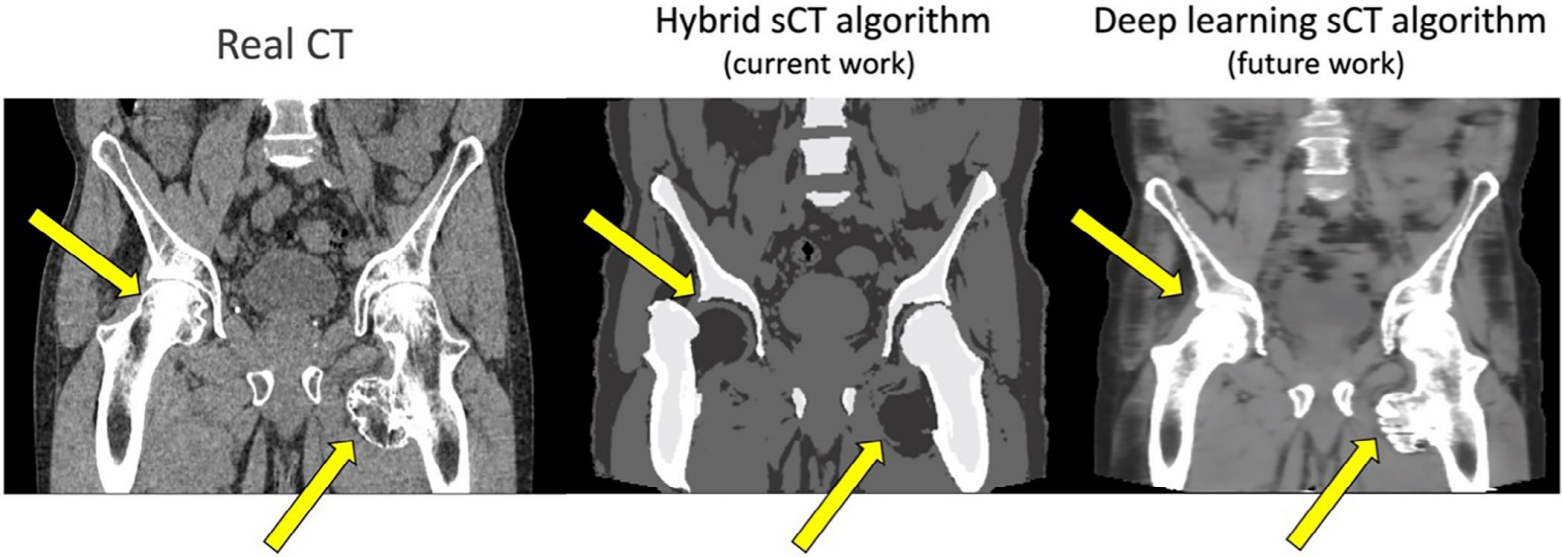
Example sagittal CT (left), sCT generated using the hybrid algorithm investigated in this study (middle), and sCT generated using the recently introduced DL‐based algorithm (right) for Patient #9 whose sCT exhibited the largest deviation in bone segmentation caused by the abnormal shape of the left femoral bone. The DL‐based algorithm appears to outperform the hybrid algorithm and will be the focus of future work. CT, computed tomography; sCT, synthetic CT.

Recent work utilizing machine learning algorithms have demonstrated excellent results in synthesizing CT from MRI.^28^, ^29^ These techniques typically utilize 2D or 3D deep neural networks that use convolution kernels to detect image features and have been utilized for medical image domain translation using generator‐only or generative adversarial network architectures.^30^ Over the last few years, improvements in deep learning architectures, model performance, and computing power have made DL methods a more common strategy for sCT reconstruction than bulk density and atlas‐based approaches. For example, after this study was performed, a DL‐based update to the commercial algorithm has been introduced. We plan to perform a comprehensive evaluation of this algorithm soon, however, a preliminary investigation demonstrated that the DL‐based algorithm was better able to identify the unique shape of the femoral bone of Patient #9, as shown in Figure 8. While this DL‐based algorithm is expected to further improve results and increase dosimetric accuracy of MR‐derived sCTs, the method presented in this work for optimizing sCT calibration curves still applies for any approach which synthesizes CT from MRI and may exhibit disagreement in HU values between sCTs and CTs. When it comes to institutional adoption of DL sCT algorithms, an optimal solution to improve model agreement in local patient cohorts would be to allow users to finetune DL models based on local data. However, this is not currently an option for any commercial sCT platform. Therefore, the proposed sCT calibration curve technique allows clinical users to tune the sCT‐based procedure to their local datasets when finetuning the model is not possible.

In addition to treatment planning, online image guidance is a critical component of the radiation therapy workflow. Traditional linacs often utilize onboard kV or MV imaging^31^, ^32^ while CyberKnife systems utilize orthogonal kV pairs^33^ to align to anatomical landmarks or implanted fiducial markers. In the context of MR‐only radiotherapy to the prostate, groups have reported on both soft tissue‐ and fiducial‐based^34^ image guidance. For example, Wyatt et al. described the successful clinical implementation of MR‐only radiotherapy using conebeam CT (CBCT)‐to‐sCT matching using soft tissue on a traditional C‐arm linear accelerator.^35^ They reported no statistical difference between matching CBCT‐to‐CT and CBCT‐to‐sCT, demonstrating that MR‐only prostate radiotherapy can be delivered safely by matching to soft tissue. Fiducial visualization, on the other hand, is more challenging because fiducial markers are typically made of high‐density material (e.g., gold) and appear as signal voids on conventional MRI sequences.^36^ Therefore, the location of most fiducial markers is not apparent on reconstructed sCT images. To mitigate this challenge, our group recently evaluated the accuracy of a novel artificial fiducial insertion method that allows for fiducial tracking of MR‐only generated sCTs in the CyberKnife system.^37^ In this work, we demonstrated that fiducial markers could be inserted into sCTs and used for fiducial tracking on the CyberKnife system, providing clinically acceptable setup accuracy with similar total targeting error to the CT‐based standard. In summary, it is conceivable that an MRI‐only based framework is possible through using the sCT calibration curve for optimal dose calculation in the treatment planning stage (as proposed in this work) and the soft tissue‐based or fiducial‐based tracking approaches for online image guidance (as recently reported).

When commissioning a sCT dose calculation framework in practice, a judicious comparison of the sCT HU values is imperative. While this work was limited to 10 patients, the proposed population‐based corrected CT density curve may benefit from a larger database of CT images, with the specific number of datasets depending on the complexity of sCT reconstruction approach and patient population (e.g., number of disease sites covered by the algorithm, presence/absence of implanted devices, etc.). Once a sCT calibration curve is generated, it should be validated in several test datasets which are representative of the patient population (e.g., male/female, under‐/overweight, etc.) who will undergo MR‐only planning using the optimized sCT calibration curve for dose calculation to ensure generalizability. Furthermore, separate disease site‐specific CT calibration curves may be required depending on the algorithm (e.g., this method should be validated separately on pelvis vs. brain sCT reconstruction algorithms). Different MRI systems and field strengths should also be investigated separately, as previous studies have documented T1 value variability across different MRI scanners, coils, and even pre‐ versus post‐system upgrades.^38^ However, these differences have yet to be investigated in the context of MR‐based sCT generation for radiotherapy treatment planning and is an important area of future research as the field moves towards widespread clinical implementation of sCT.

## 5 CONCLUSIONS

MRI‐derived sCT using an optimized CT calibration curve shows good dosimetric agreement with conventional CT simulation, demonstrating the feasibility of using MRI‐derived sCT for prostate SBRT treatment planning. Accurate delineation of bone remains a challenge for reconstruction of MRI‐based sCT and improvements in this space could potentially allow for the implementation of an MRI‐only framework.

## AUTHOR CONTRIBUTIONS

Jessica Scholey First author responsible for study design and execution. Completed image quality analysis/quantification and dosimetric analysis of study and wrote manuscript. Tomi Nano Second author responsible for study design and execution. Assisted with image quality analysis and quantification. Kamal Singhrao Third author responsible for study design and execution. Assisted with image quality analysis and quantification. Osama Mohamad Aided with patient selection and provided medical expertise on MRI imaging for prostate cancer. Lisa Singer Aided with patient selection and provided medical expertise on MRI imaging. Peder Larson Co‐senior author responsible for validation of study design, providing resources for study completion and validation of study results. Martina Descovich Co‐senior author responsible for validation of study design, providing resources for study completion and validation of study results.

## CONFLICT OF INTEREST STATEMENT

The authors declare no conflicts of interest.
